# High mechanical property silk produced by transgenic silkworms expressing the *Drosophila* Dumpy

**DOI:** 10.3389/fbioe.2024.1359587

**Published:** 2024-02-12

**Authors:** Xiangping Dai, Xiaogang Ye, Liangen Shi, Shihua Yu, Xinqiu Wang, Boxiong Zhong

**Affiliations:** ^1^ Institute of Silkworm and Bee Research, College of Animal Sciences, Zhejiang University, Hangzhou, China; ^2^ Key Laboratory of Silkworm and Bee Resource Utilization and Innovation of Zhejiang Province, Hangzhou, China; ^3^ Institute of Applied Bioresource Research, College of Animal Sciences, Zhejiang University, Hangzhou, China

**Keywords:** mechanical properties, transgenic silkworms, *Drosophila* Dumpy, piggyBac, transgenic silks

## Abstract

Extensive research has been conducted on utilizing transgenic silkworms and their natural spinning apparatus to produce high-performance spider silk fibers. However, research on using non-spider biological proteins to optimize the molecular structure of silk protein and improve the mechanical performance of silk fibers is still relatively scarce. Dumpy, a massive extracellular matrix polypeptide, is essential for preserving the shape and structural integrity of the insect cuticle due to its remarkable tension and elasticity. Here, we constructed two transgenic donor plasmids containing the fusion genes of *FibH-Dumpy* and *FibL-Dumpy*. The results indicated the successful integration of two exogenous gene expression cassettes, driven by endogenous promoters, into the silkworm genome using piggyBac-mediated transgenic technology. Secondary structure analysis revealed a 16.7% and 13.6% increase in the β-sheet content of transgenic silks compared to wild-type (WT) silk fibers. Mechanical testing demonstrated that, compared to the WT, HDUY and LDUY transgenic silk fibers exhibited respective increases of 39.54% and 21.45% in maximum stress, 44.43% and 45.02% in toughness, and 24.91% and 28.51% in elastic recovery rate. These findings suggest that *Drosophila* Dumpy significantly enhanced the mechanical properties of silk, positioning it as an excellent candidate for the development of extraordinary-performance fibers. This study provides rich inspiration for using other biological proteins to construct high-performance silk fibers and expands the possibilities for designing and applying novel biomaterials.

## Introduction

The silkworm (*Bombyx mori*), has been widely acknowledged as an economically significant domesticated insect and an ideal model organism for Lepidoptera (Duan et al., 2010; Zhang et al., 2021). Silk spun by silkworms is garnering increased attention due to its extraordinary mechanical characteristics, ideal biocompatibility and controllable solubility (Qiu et al., 2019). These characteristics have endowed silk fibers with a wide range of applications in fields including textiles, medicine, and the military (Tao et al., 2012). Silkworm silk consists of two main types of proteins, fibroins and sericins, which are synthesized and secreted by the posterior silk gland (PSG) and the middle silk gland (MSG), respectively. The fibroins include the fibroin heavy chain (FibH), the fibroin light chain (FibL), and the fibrohexamerin (P25), with molecular weights of approximately 350 kDa, 26 kDa, and 30 kDa, respectively (Chevillard et al., 1986; Zhou et al., 2000). It is generally believed that specific hexapeptide repetitive units (GAGAGS) and the large molecular weight of the *FibH* gene are the determining factors of silk mechanical performance (Tanaka et al., 1999).

During the past decades, significant advancements in genetic manipulation approaches, such as piggyBac-mediated transposon technology, zinc finger nucleases, transcription activator-like effector nucleases (TALENs), and clustered regularly interspaced short-palindromic repeats (CRISPR) systems, have been successfully utilized to generate foreign proteins in *B. mori* (Takasu et al., 2010; Ma et al., 2012; Wang et al., 2013a). For instance, by utilizing the piggyBac transposon approach, synthetic spider silk genes of varying lengths were randomly inserted into the genome of silkworm, resulting in improved mechanical properties in the chimeric fibers (You et al., 2018; Tang et al., 2021). Researchers employed the native FibH promoter to express the recombinant spider protein, and the maximum stress reached 600 MPa in composite fibers (Kuwana et al., 2014). In addition, previous research has demonstrated that replacement of the *FibH* gene with the synthesized *MaSp1* spider silk gene through TALEN-mediated homology-directed repair has led to enhanced toughness and extensibility of chimeric fiber (Xu et al., 2018). These studies indicated that introducing exogenous spider silk proteins into silk is an effective approach for enhancing the mechanical properties of silk fibers. However, in addition to spider silk protein, various other proteins with excellent performance and unique biological functions have emerged during long-term evolution (Zhao et al., 2023). Therefore, it is meaningful to investigate silk performance improvement using these natural proteins as an alternative to spider silk proteins.

The *Dumpy* locus is genetically complex, and the genetic fine structure map was published in the middle of the last century (Sederoff, 1967). However, detailed information about the modular structure of Dumpy was first described in *Drosophila melanogaster* by Ross MacIntyre in 2000 (Wilkin et al., 2000). *Drosophila* Dumpy includes 78 coding exons, encoding a massive 2.5 MDa extracellular matrix polypeptide consisting of more than 300 epidermal growth factor-like (EGF) modules and a four-cysteine module termed dwarf (DPY) (Carmon et al., 2010a). Notably, a considerable portion of the Dumpy consists mainly of consecutive repeats of a three module EGF–DPY–EGF unit, and the organization of these repeats was found to be stable among insects (Carmon et al., 2007). Besides, through the examination of Dumpy’s cysteine-spacing pattern, a super repeat comprising of six contiguous EGF-DPY-EGF units was identified, occurring a total of thirteen times across Dumpy. The high-order structural repeat units played an essential role in tissue morphogenesis and maintenance of mechanical tension at sites (Wilkin et al., 2000). Moreover, the repeat regions and EGF–EGF linkers abundant in serine and proline residues in the Dumpy fiber confer flexibility and elasticity to maintain the shape and structural integrity of the cuticle in response to stress (Carmon et al., 2010b; Cho et al., 2021). Based on the above characteristics, we speculate that the *Dumpy* gene may be an excellent candidate for developing new biomaterials with superior mechanical properties. The high molecular weight and repeating modular structure shared by Dumpy and FibH make the genetically modified silkworm an excellent bioreactor for the production of re-Dumpy silk protein.

In the present study, we focused on the *Drosophila* Dumpy protein and constructed two transgenic donor plasmids incorporating fusion genes of *FibH-Dumpy* and *FibL-Dumpy*. Our results indicated that two exogenous gene expression cassettes driven by endogenous promoters were successfully integrated into the silkworm genome using piggyBac-mediated transgenic technology. Western blot analysis confirmed the successful expression of re-Dumpy protein in the posterior silk gland, subsequently secreted into the cocoon shell. Then, we integrated data regarding secondary structures and tensile testing analysis to evaluate the mechanical properties of chimeric fibers. The results demonstrated that the transgenic silk fibers had higher strength, measuring 248.83 MPa and 216.57 MPa, which represented enhancements of 39.54% and 21.45%, respectively, compared to wild-type (*Lan 10*) fibers. Additionally, the transformed silkworm silk fibers displayed significant increases in β-sheet content, Young’s modulus, toughness and elasticity. All data collectively suggest that Dumpy holds promise as a candidate target for developing extraordinarily high-performance silk materials.

## Materials and methods

### Animals

The non-diapausing silkworm strain *Lan 10* (preserved in the silkworm Genetics Laboratory of Zhejiang University), was used in the study. The larvae were raised on fresh mulberry leaves under standard conditions.

### PiggyBac vector construction

The repetitive unit sequence of *Dumpy* from *D. melanogaster* (accession number: CG33196) was optimized based on the codon preference of silk proteins, and has been deposited in the GenBank database (accession number: OR224864). The repetitive unit sequence of Dumpy with a His-tag at the C-terminus was synthesized by GENEWIZ (Suzhou, China). The Dumpy sequences were then sub-cloned into pUC57 (TaKaRa, China) to produce pUC57-HDumpy and pUC57-LDumpy vectors, which contained AgeI/NheI and XmaI/NheI restriction enzyme sites, respectively. The targeted fragment of the Dumpy gene from pUC57-HDumpy was digested with AgeI/NheI enzymes. The purified fragment was then integrated into the pBac [FibH-IE1-DsRed] vector (preserved in our laboratory) to generate the final transgenic expression vector pBac [FibH-Dumpy-IE1-DsRed]. In the same way, the final donor vector pBac [FibL-Dumpy-IE1-DsRed] was also successfully constructed. The sequence information contained in the plasmids was provided as Supplementary Datas S1, S2.

### Screening of positive individuals

The nondiapause silkworm *Lan 10*, was used for the microinjection experiment. The piggyBac transposition system was performed as previously described (Wu et al., 2021). Briefly, the piggyBac donor plasmid [FibH-Dumpy-IE1-DsRed]/[FibL-Dumpy-IE1-DsRed] and helper plasmid were mixed at a final concentration of 200 ng/μL. The plasmid mixtures were then microinjected into 800 preblastoderm embryos. The G0 silkworm larvae were raised under standard conditions to the moth stage. The surviving G0 moths were mated to WT moths to obtain the G1 broods. To select the positive transgenic individuals, the G1 silkworms with ubiquitous red fluorescence were screened using fluorescence microscopy (Olympus, Japan).

### Inverse PCR

Inverse PCR assay was performed as described previously (Ye et al., 2023). Genomic DNA of G2 DsRed-positive larvae was completely digested with the Sau3AI restriction enzyme and circularized by T4 DNA ligase (TaKaRa, China). The ligated fragments were directly used as PCR amplification templates. The purified PCR products were sub-cloned into the pMD19-T vector (TaKaRa, China) followed by sequencing. The insertion location was further analyzed against the silkworm genome database (http://sgp.dna.affrc.go.jp/KAIKObase/). All related primer sequences are listed in Supplementary Table S1.

### Quantitative real-time PCR

Total RNA was extracted from the PSGs dissected from the third day of the fifth instar with TRIzol Reagent (Invitrogen, United States) as previously described (You et al., 2017). Quantitative real-time PCR (qRT-PCR) assay was performed utilizing an ABI Stepone PCR Detection System (BIO-RAD, United States) with SYBR qPCR Master Mix (Vazyme, China). All primers are presented in Supplementary Table S1. Relative gene expression level was analyzed using the 2^−ΔΔCT^ method as previously described (Livak and Schmittgen, 2001). The measurements of transcription level were quantitated in three replicates and normalized to the *Bmrp49* gene (accession number: NM_001098282).

### Western blotting

Extraction of the cocoon shell protein was performed as previously described with slight modifications (Wang et al., 2013b). Cocoons shell from WT and transgenic individuals were cut into small pieces and grind them into powder using the tissue grinder (Jingxin, China). 50 mg cocoon powder samples were immersed in 1 mL SDS-protein extraction solution overnight at 37°C. Protein samples were then subjected to SDS-PAGE (4%–15%), followed by staining with Fast Silver Stain Kit (Beyotime, China). For western blotting, the membranes were incubated with an anti-His antibody (1:3000, Sangon, China) and an anti-re-Dumpy specific peptide antibody (1: 1000, GenScript, China) as the primary antibodies. The specific polyclonal re-Dumpy antibody was raised in rabbits against the peptide “CRPAPPPEPTQSEYV” (GenScript, China). The signals were detected using the chemiluminescence imaging system (Clinx, China).

### SEM observation and FTIR spectroscopy analysis

The sub-structural morphology of the cocoon shells were investigated using field emission-scanning electron microscopy (SEM, SU8010, Hitachi, Japan). Fourier-transform infrared spectroscopy (FTIR) analysis was performed as previously described with minor modifications (Zhao et al., 2021). First, the cocoons were soaked in a 0.5% Na_2_CO_3_ solution to degum in 85°C water bath. After washing with deionized water three times, the degummed cocoons were air-dried overnight. Next, the dried samples were crushed into powder and mixed with KBr in a ratio of 1:100. The mixtures were further analyzed using a NICOLET iS50FT-IR instrument (Thermo Scientific, United States). Spectral data analysis was performed using OMNIC and Origin 9.1 software.

### Mechanical testing of silk fibers

Five cocoons from WT and transgenic silkworm strains were randomly chosen and three monofilaments were taken from each cocoon for the measurement of mechanical properties (*n* = 15). Single fibers were acquired according to a previous study with appropriate modifications (Zhao et al., 2007). Individual fibers were tested for maximum stress, maximum strain, Young’s modulus, and toughness. The diameter of each silk fiber was determined by taking an average of five measurements using a digital microscope (VHX-600, Keyence, Japan). The cocoon silks were then subjected to a tensile test using an AGS-J Universal Test instrument (Shimadzu, Japan) equipped with a standard 5 N load cell at a strain rate of 2 mm/min until the fiber broke. Data analysis was performed with Origin 9.1 software.

The elastic recovery rate assay was conducted according to the literature (Zhao et al., 2023). Briefly, the elastic recovery of silk fibers at constant elongation was tested by an AGS-J Universal Test instrument (Shimadzu, Japan) under standard conditions. The test conditions included a sample holding distance of 30 mm, a stretching speed of 5 mm/min, 10 cycle times, and a fixed elongation value of 10%. Each silk sample underwent 6 tests, and the average value was recorded. The formula for calculating the elastic recovery rate was R = (L−L’)/L ×100%. R represents the elastic recovery rate, L represents the total elongation and L’ represents the residual elongation.

### Statistical analysis

The experimental data were analyzed using Student’s *t*-test. The significance levels were represented as *p < 0.05, **p < 0.01, ***p < 0.001, and n.s. for *p* > 0.05. The data are reported as the mean ± SEM for at least triplicate experiments.

## Results

### Generation of transgenic silkworms

**FIGURE 1:**
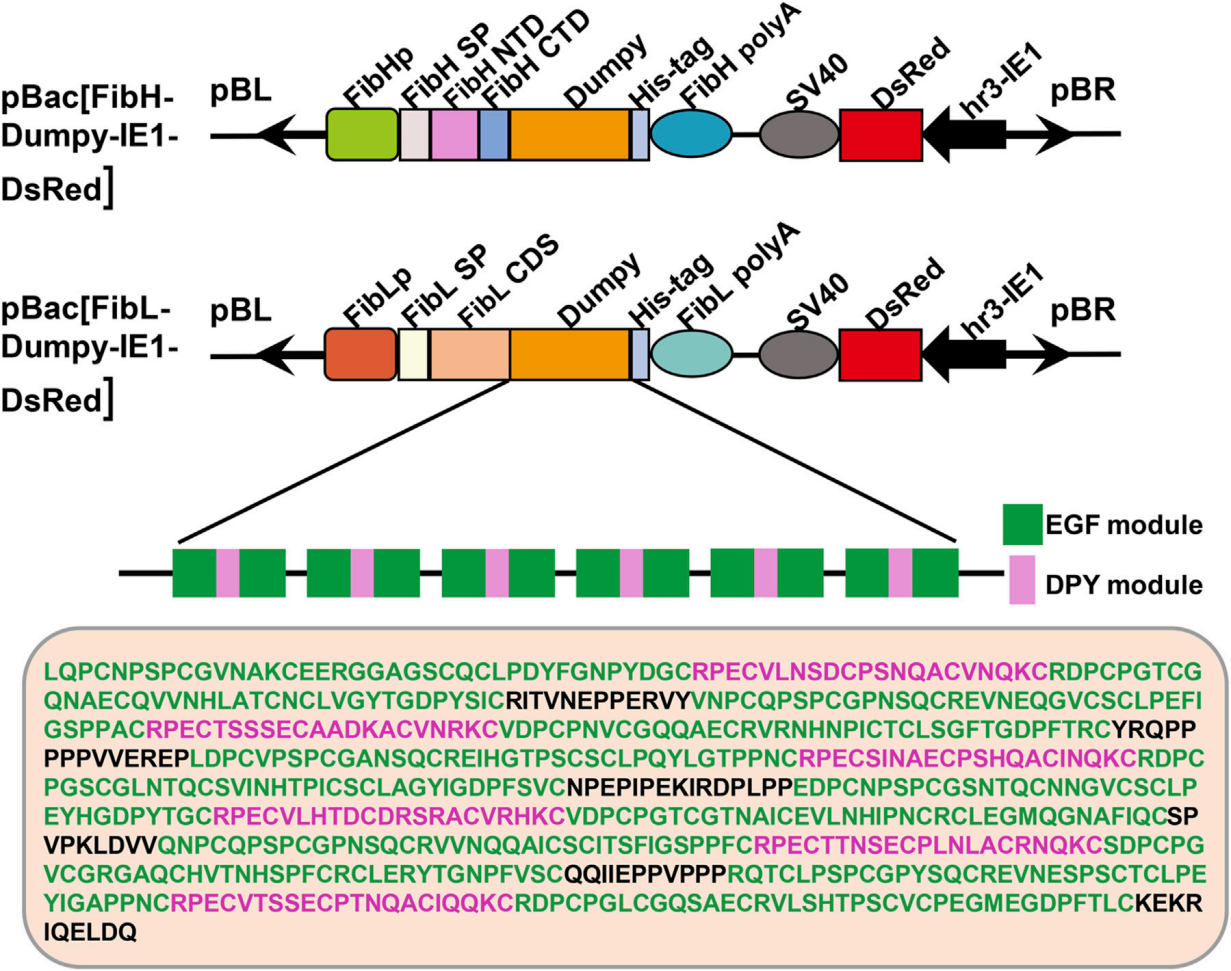
Schematic representation of the transgenic vectors. Structures of the pBac [FibH-Dumpy-IE1-DsRed] vector and pBac [FibL-Dumpy-IE1-DsRed] vector. The key elements are indicated with different colors. Orange represents the six consecutive EGF-DPY-EGF units (a super repeat) of *Drosophila* Dumpy (639 aa). The marker gene *DsRed* was employed to screen positive transgenic silkworms. EGF, epidermal growth factor module; DPY, a four-cysteine module.

The sequence of Dumpy is highly regular, consisting predominantly of consecutive repeats of a three-module EGF-DPY-EGF unit, which contributes to the maintenance of mechanical tension at sites under stress in *Drosophila* (Wilkin et al., 2000). To ascertain the mechanical properties of recombinant Dumpy silk fiber, we constructed two transgenic donor plasmids in this study (Figure 1). One plasmid (FibH-Dumpy-IE1-DsRed) contained the *FibH* N-terminal domain, *FibH* C-terminal domain and six consecutive EGF–DPY–EGF units (a super repeat) of *Dumpy* (1917 bp) with a His-tag, driven by an endogenous *FibH* promoter. The other plasmid (FibL-Dumpy-IE1-DsRed) contained the coding sequence of *FibL* and identical repetitive unit sequences of *Dumpy* (1917 bp) with a His-tag, driven by a native *FibL* promoter. The red fluorescence expression induced by the, *IE1* promoter facilitated the screening of positive individuals (Figure 1).

**FIGURE 2:**
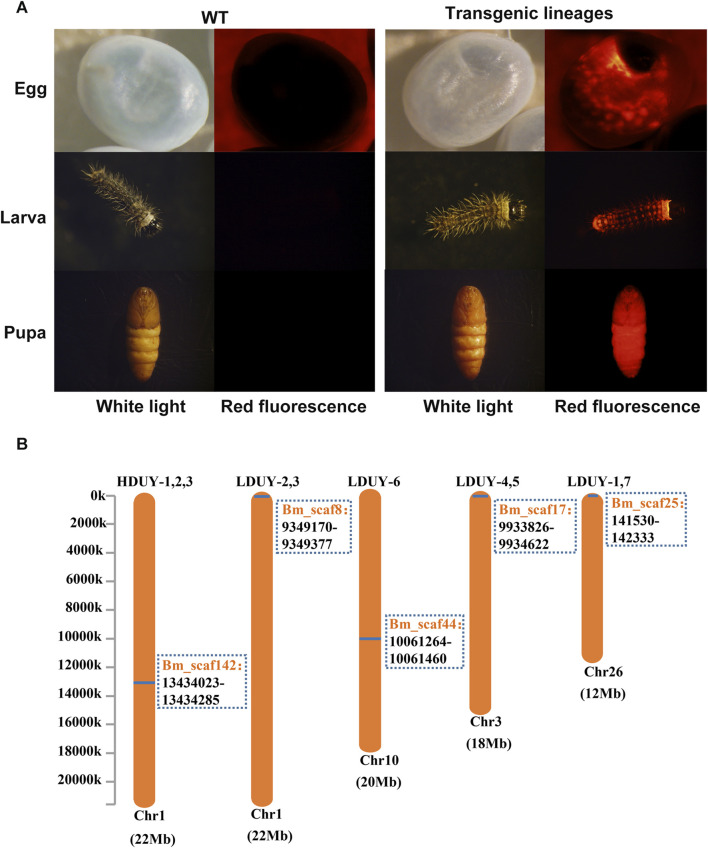
Generation of transgenic positive silkworms. **(A)** Specific red fluorescence of positive transgenic strains at different developmental stages. G1 egg, larva and pupa of the WT and positive-transgene silkworms were observed under white light and red fluorescence. **(B)** The insertion location of the HDUY and LDUY strains on the chromosome. The inverse PCR primers are listed in Supplementary Table S1.

To obtain transgenic silkworms, a mixture of donor and helper plasmids was injected into 800 preblastoderm embryos. The hatched eggs were raised to the moth stage, and crossed with WT individuals. Consequently, we obtained 3 positive G1 broods from 51 broods of the FibH-Dumpy-IE1-DsRed group under fluorescence microscopy and designated them HDUY-1, -2, and -3. In parallel, we obtained 7 positive G1 broods from 278 broods of the FibL-Dumpy-IE1-DsRed group under the fluorescence microscopy and named LDUY-1∼7. The transformation efficiency in the HDUY and LDUY groups was 5.9% and 2.5%, respectively (Supplementary Table S2). Additionally, a ubiquitous red fluorescence signal was detected throughout the developmental stages in the HDUY and LDUY positive silkworm individuals (Figure 2A).

### *Dumpy* gene inserted into the silkworm genome

Previous researches have shown that piggyBac-mediated gene transposition predominantly occurs at TTAA sites in the genome, displaying both insertional preferences and random characteristics (Wilson et al., 2007; Galvan et al., 2009). Therefore, we conducted inverse PCR to further identify the specific insertion locations in transgenic lineages. Genomic DNA was extracted from the PSG tissues of G2 DsRed-positive broods (3 broods from donor FibH-Dumpy-IE1-DsRed and 7 broods from donor FibL-Dumpy-IE1-DsRed), digested with the restriction enzyme Sau3AI and further confirmed by inverse PCR. The result indicated that just one insertion site was detected within the intronic region of chromosome 1 in the HDUY transgenic strain (Figure 2B; Supplementary Table S3). However, the LDUY transgenic silkworms possessed 4 different integration sites in the silkworm genome. Among them, LDUY-6 exhibited a single insertion site situated in the intronic region of chromosome 10. LDUY-2 and LDUY-3 shared a common insertion site situated in the intergenic region of chromosome 1. Meanwhile, LDUY-4/-5 and LDUY-1/-7 exhibited identical insert positions, located in the intronic region of chromosome 3 and chromosome 26, respectively (Figure 2B). In addition, no notable discrepancies in phenotype or behavioral abnormalities were detected between WT and HDUY/LDUY transgenic lines during the developmental period under standard feeding conditions. HDUY-1 and LDUY-6 lines exhibited stronger red fluorescence expression and were selected to establish stable transgenic strains for subsequent experiments.

### Dumpy protein expressed in transgenic silkworms

**FIGURE 3:**
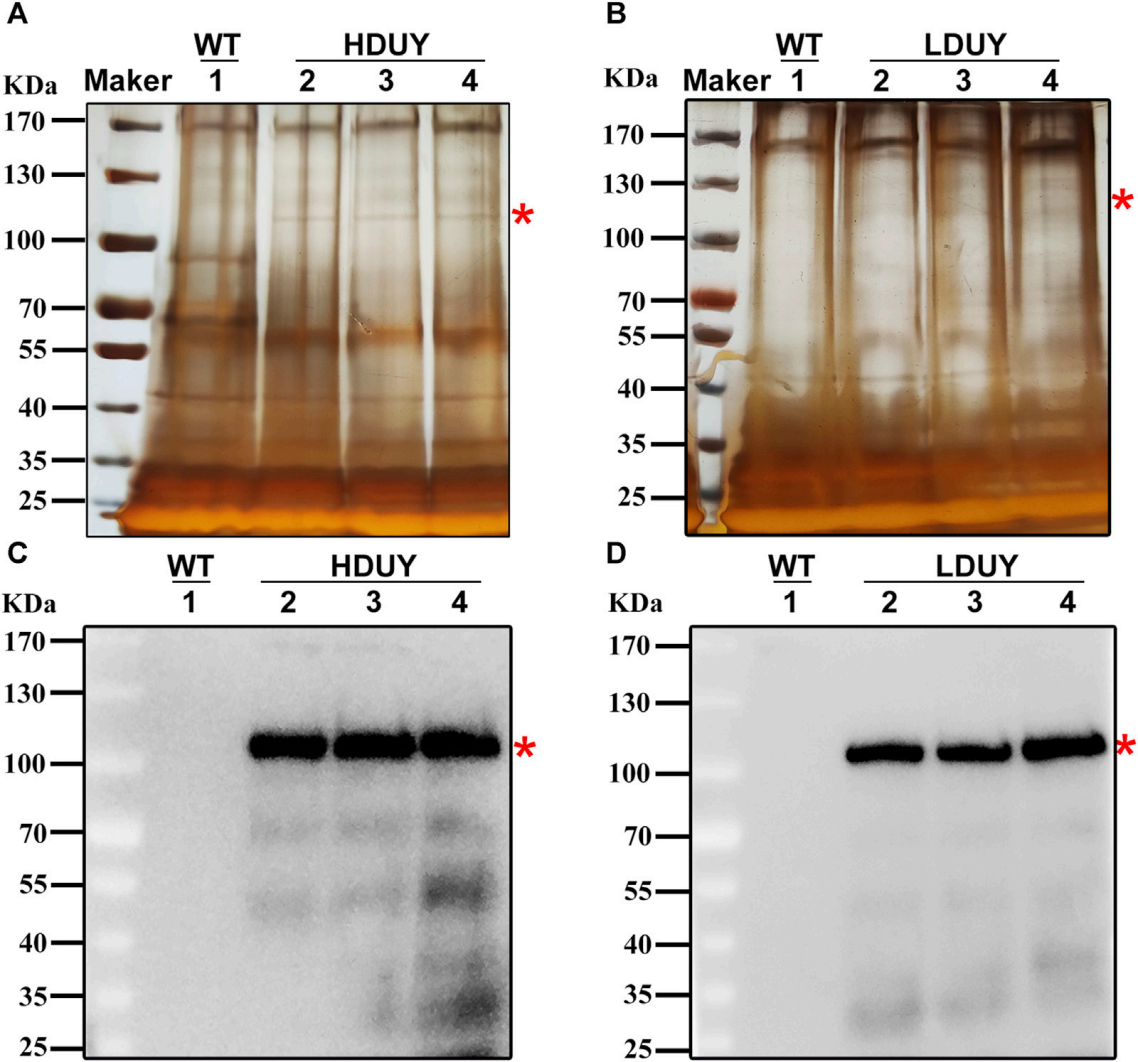
Dumpy expression in cocoon shells of transgenic silkworms. Protein extracted from the WT and transgenic cocoons was subjected to SDS-PAGE analysis followed by silver staining **(A,B)** and Western blotting **(C,D)**. 1: silkworm *Lan 10* (WT). 2, 3, and 4: three different individuals from HDUY or LDUY transgenic lines. Immunoblot analysis was performed using an anti-His antibody. The predicted target proteins are marked with red asterisks. The molecular masses of the protein standards are shown on the left.

Although red fluorescence expression was detected in genetically modified silkworms, it remains unclear whether the re-Dumpy protein was successfully expressed in PSG and secreted into the cocoon shell. Therefore, we dissected PSG tissues from the third day of the fifth instar larvae and conducted qRT-PCR experiments to investigate the transcript level of exogenous *Dumpy*. The profiles showed that the signals were detected in PSGs of the HDUY and LDUY strains but not the WT (Supplementary Figure S1A). Of note, the *Dumpy* gene in the HDUY strain was expressed at a higher level than in LDUY. Then, we extracted the cocoon shell proteins as described previously (You et al., 2018), and performed SDS-PAGE and Western blot analysis to further confirm the expression of Dumpy. Silver staining analysis revealed a single band above 100 kDa, which appeared slightly larger than the predicted size of the target protein. (HDUY, 97 kDa; LDUY, 93 kDa) (Figures 3A, B). Furthermore, immunoblot analysis demonstrated no bands in the WT individuals but demonstrated protein bands in transgenic individuals which were also larger than the predicted molecular weights, further confirming that the re-Dumpy proteins were successfully expressed and secreted into the transgenic silkworm cocoons (Figures 3C, D; Supplementary Figures S1B, C). The molecular weights of these bands were larger than the predicted sizes, which was likely caused by posttranslational modifications.

### Dumpy silk fibers increased the content of β-sheet structure

**FIGURE 4:**
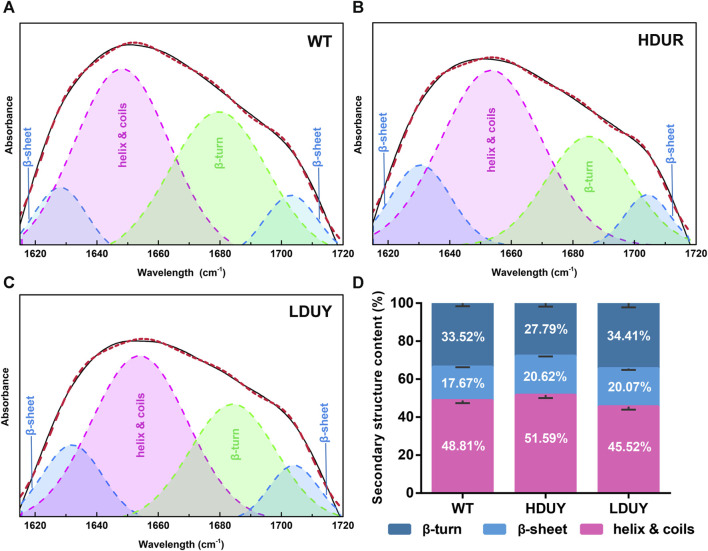
Secondary structure analysis of silk fibers. Deconvolutional results of amide I band of silk fibers from the WT **(A)**, HDUY **(B)**, and LDUY **(C)**. **(D)** Comparison of the secondary structure contents of cocoon silks from the WT and two transgenic groups. Vertical bars represent the mean ± SEM (*n* = 3).

The transgenic silkworms were able to synthesize and secrete the re-Dumpy protein into the silk cocoon shell. To determine the effect of transformation on silk fibrillogenesis, silk fibers and cocoons were collected from two transgenic lines for further analysis. First, morphological observation and scanning electron microscopy were performed on the silkworm cocoons. The results exhibited no obvious differences among the WT, HDUY and LDUY groups (Supplementary Figures S2A, B). Additionally, no notable disparities in cocoon weight or cocoon shell weight were observed in the transgenic groups compared to the control group (Supplementary Figures S2C, D). Subsequently, the secondary structure composition of the degummed silk fibers was determined using infrared spectroscopy, specifically focusing on the amide I region (1,600–1,700 cm^−1^) (Supplementary Figure S3A). We determined the characteristic absorption peaks of each secondary structure in silk through amide I band extraction and spectrum deconvolution (Figures 4A–C). According to previous studies, the peaks located in the regions of 1,615–645 cm^−1^ and 1,690–1,700 cm^−1^ were attributed to the β-sheet structure, while the peak situated at 1,680–1,690 cm^−1^ was assigned to the β-turn component. Moreover, the spectral contribution between 1,649 and 1,659 cm^−1^ was assigned as a helix or coil structure (Zhao et al., 2021). The results of spectrum deconvolution provided an estimated content of 51.59% helix/coils and 27.79% β-turn in HDUY silk fibers, 45.52% helix/coils and 34.41% β-turn in LDUY silk fibers, and 48.81% helix/coils and 33.52% β-turn in WT silk fibers. (Figure 4D; Supplementary Table S4). Moreover, the β-sheet structure content, corresponding to 20.62% and 20.07% of amide I absorbance in the HUDY and LDUY silks, increased by 16.69% and 13.58% compared to that of the WT (Figure 4D; Supplementary Figure S3B).

### Dumpy enhanced mechanical properties of transgenic silk fibers

**FIGURE 5:**
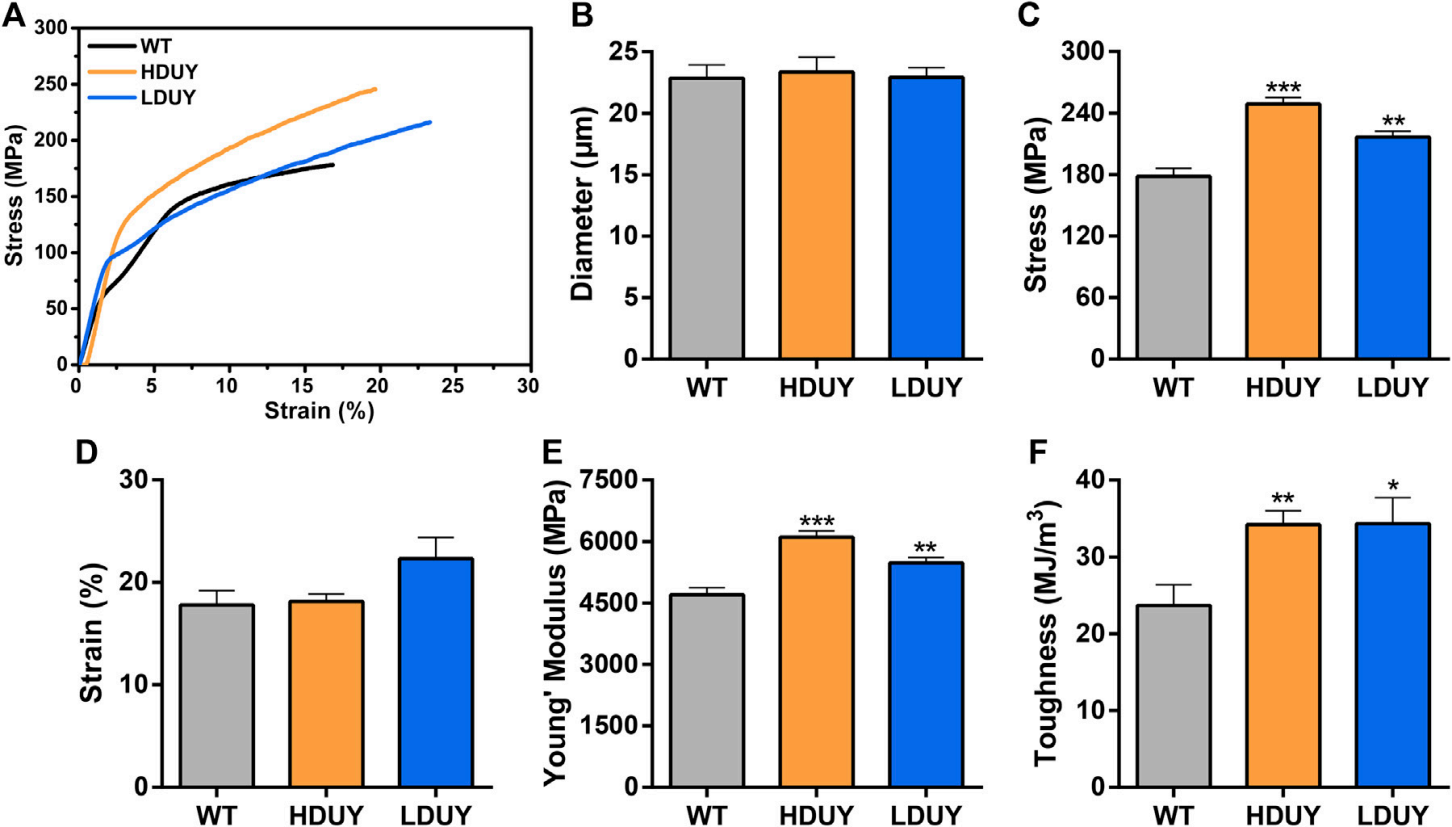
Mechanical properties of the composite silk fibers. **(A)** Stress-strain curve of silk fibers from the WT (black), HDUY (yellow) and LDUY (blue). The curve represents the average of 15 single experiment curves. **(B–F)** Comparisons of the diameter, maximum stress, maximum strain, Young’s modulus, and toughness of silk fibers. *p < 0.05, **p < 0.01 and ***p < 0.001. Error bar, SEM.

**FIGURE 6:**
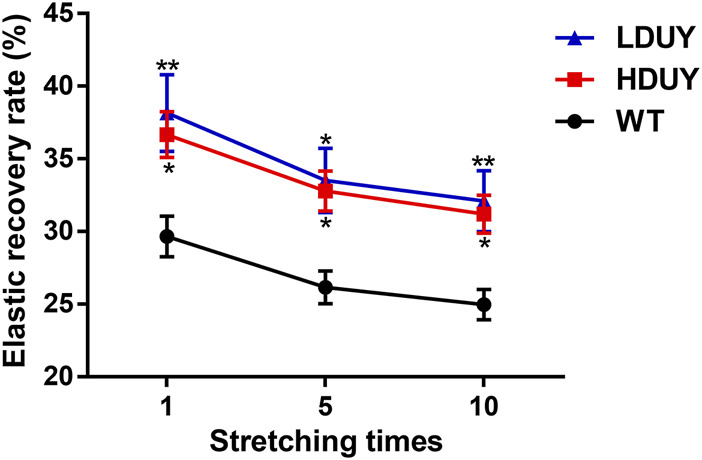
Elastic recovery rate test of the composite silk fibers. The elastic recovery property of silk fibers was tested at a constant elongation value (10%). The elastic property for each kind of fiber was determined by taking an average of six. 1, 5, and 10 represented stretching times. The elastic recovery rates for the WT, HDUY and LDUY fibers are shown in black, red and blue, respectively. *p < 0.05, **p < 0.01. Error bar, SEM.

The content of secondary structures in the silk fibers exhibited clear differences between WT and transgenic individuals, implying that silk mechanical performance probably occur alterations. Single silk fibers were reeled from the cocoons as previously described (You et al., 2018). Then, mechanical testing was conducted on the transgenic silk fibers, and their mechanical properties relative to the WT were measured under identical conditions. The average stress-strain curve demonstrated a significant enhancement in the breaking stress of the silk fibers in both the HDUY and LDUY groups, suggesting that the fibers would exhibit greater strength compared to the WT group (Figure 5A). Next, we investigated four mechanical parameters (maximum stress, maximum strain, Young’s modulus, and toughness) of the single silk fibers (Table 1). The transgenic silk fibers had higher maximum stress, reaching 248.83 MPa and 216.57 MPa, which was enhanced by 39.54% and 21.45%, respectively, compared to WT fibers, which had a maximum stress of 178.32 MPa (Figures 5B, C). However, the composite silk fibers from the HDUY and LDUY groups exhibited a slight increase in maximum strain (Figure 5D). Furthermore, the average value of Young’s modulus from the two transgenic strains was 6,102.98 MPa and 5,480.32 MPa, enhanced by 29.79% and 16.55%, respectively, compared to WT fibers (Figure 5E). Finally, the Dumpy chimeric fibers showed toughness of 34.20 MJ/m^3^ and 34.34 MJ/m^3^, demonstrating that they were tougher than the WT fiber, which had a toughness of 23.68 MJ/m^3^ (Figure 5F). Subsequently, the elastic recovery rate of the silk fibers was tested with a constant elongation rate of 10%, and the results are presented in Figure 6; Table 2. In a single tensile recovery test, the elastic recovery rates of WT, HDUY and LDUY silk were 29.66%, 36.66%, and 38.15%, respectively. Notably, the elastic recovery rates of HDUY and LDUY silk were 23.60% and 28.62% higher than the control, respectively. In the cyclic tensile recovery test, with the increase in the stretching number, the ability of tensile recovery in fibers was gradually weakened. By the 10th time, the elastic recovery rates decreased to 24.97%, 31.19%, and 32.09% in the WT, HDUY and LDUY groups, respectively. Compared with the WT, the elastic recovery rates of transgenic silkworm silks were increased by 24.91% and 28.51%, respectively. Taken together, these findings indicated that the re-Dumpy silk fibers had significantly increased mechanical performance.

**TABLE 1:** Mechanical properties of WT and transgenic silk fibers.

Silkworm strains	Maximum stress (MPa)	Maximum strain (%)	Young’modulus (MPa)	Toughness (MJ/m^3^)
WT	178.32 ± 29.68	17.78 ± 5.47	4,702.16 ± 654.52	23.68 ± 10.42
HDUY	248.83 ± 24.50	18.15 ± 2.79	6,102.98 ± 586.04	34.20 ± 6.97
LDUY	216.57 ± 22.67	22.32 ± 8.02	5,480.32 ± 508.42	34.34 ± 13.04

**TABLE 2:** Resilience test of the composite silk fibers.

Stretch ratio	10%		
Stretching times	Elastic recovery rate of WT (%)	Elastic recovery rate of HDUY (%)	Elastic recovery rate of LDUY (%)
1	29.66 ± 3.41	36.66 ± 3.85	38.15 ± 6.44
2	28.08 ± 2.99	34.77 ± 3.47	35.85 ± 5.82
3	27.15 ± 2.89	33.83 ± 3.41	34.82 ± 5.88
4	26.63 ± 2.81	33.18 ± 3.39	34.38 ± 3.85
5	26.15 ± 2.76	32.78 ± 3.36	33.50 ± 3.41
6	25.88 ± 2.72	32.33 ± 2.36	33.25 ± 5.48
7	25.59 ± 2.68	31.98 ± 3.29	32.84 ± 3.42
8	25.39 ± 2.68	31.72 ± 2.21	32.63 ± 2.49
9	25.19 ± 2.59	31.47 ± 3.21	32.31 ± 3.29
10	24.97 ± 2.57	31.19 ± 3.19	32.09 ± 2.57

## Discussion

Natural biological proteins afford a wealth of inspiration for constructing high-performance materials with wide-ranging applications. Dumpy is a gigantic extracellular matrix polypeptide with high tension and elasticity and is highly conserved in the evolution of insects (Carmon et al., 2007). In the study, we described the successful transfer of fusion genes of *FibH-Dumpy* and *FibL-Dumpy* into the silkworm genome using piggyBac-mediated transgenic technology. Screening of *DsRed* fluorescent marker genes, combined with inverse PCR and insertion site analysis, confirmed that the *Dumpy* fusion genes had been successfully incorporated into the silkworm genome. Western blot analysis indicated successful expression and secretion of the re-Dumpy protein in the composite silk. Moreover, our experimental analyses demonstrated that the genetically modified silk fiber had significantly increased β-sheet content, and higher strength, extensibility and elasticity compared to that of the WT, suggesting that Dumpy is a promising candidate target for developing extraordinary-performance silk materials.

We introduced two plasmids containing six EGF -DPY-EGF repeats of Dumpy into the silkworm genome and obtained two transgenic lines, HDUY and LDUY. The length of the target sequence was 1917 bp, and the predicted molecular weights of chimeric Dumpy were 97 kDa and 93 kDa. However, silver staining and Western blot analyses revealed that the molecular weights of the target bands exceeded the predicted sizes. We speculate that it may be caused by the posttranslational modifications. Taking into account the relatively small molecular weight of the re-Dumpy (∼100 kDa), this excellent mechanical performance will be further enhanced by integrating Dumpy protein of larger molecular weight. A previous study have found an extremely significant linear relationship between exogenous gene length and mechanical properties in composite silk fibers (You et al., 2018). Therefore, access to sufficiently long protein chains is an essential part of access to high performance protein-based silk fibers. Additionally, previous studies have shown that the improvement of extensibility and toughness of fused fibers largely depends on whether the integrated exogenous genes possess distinct secondary structures and whether the high molecular weights include repetitive units (Xu et al., 2018). Therefore, we propose that it is a feasible strategy to develop chimeric silk materials with superior performance by integrating more Dumpy repetitive structures and higher molecular weights into the silkworm genome.

The incorporation of Dumpy resulted in significant alterations to the mechanical properties of the silkworm silk fiber. Compared to the WT fibers, the average values of maximum stress, Young’s modulus, and toughness were significantly enhanced. Our tensile testing showed that the average maximum stress of WT (*Lan 10*) was 178.32 MPa, and this result was consistent with previous researches (You et al., 2018; Zhao et al., 2023). The average maximum stress from the HDUY and LDUY silks was 248.83 MPa and 216.57 MPa, enhanced by 39.54% and 21.45%, respectively, compared to the WT. However, differences among silkworm strains may potentially impact the maximum stress value. In future research endeavors, we can integrate *Dumpy* gene into the genomes of silk production specific silkworm varieties, thus generating transgenic silk fibers with even more remarkable mechanical properties. Of note, the improvement levels of the mechanical properties of the HDUY strain seems to be higher than those of the LDUY strain. We presume that this result was due to the variation in insertion sites of the foreign *Dumpy* gene on the silkworm chromosomes. Owing to the position effect, the random insertion sites of exogenous *Dumpy* genes on the genome may have influenced the *Dumpy* expression level. qRT-PCR results showed that the *mRNA* expression of the *Dumpy* gene in HDUY was significantly elevated in HDUY compared to LDUY. Western blot analysis also demonstrated higher protein expression levels in HDUY transgenic individuals. A high level of expression indicates that a greater quantity of fusion proteins, formed by the combination of Dumpy proteins and silk fibroin proteins, are able to participate in the assembly of the silk complex and formation of the secondary structure. As a result, the mechanical properties of HDUY silk were significantly enhanced.

The size and motifs of silk proteins are crucial factors that influence the mechanical properties of silk fibers (You et al., 2018). Dumpy is highly regular and consists of a continuous repetition of three modular EGF -DPY-EGF units (Wilkin et al., 2000). The high molecular weight and repeating modular structure were similar to those of FibH, presumably enhancing the mechanical properties of the protein. Previous research has shown that the main structural component in silk is the β-sheet crystals, which determines the mechanical strength of silk (Xu et al., 2022). Our secondary structure analysis revealed a significant enhancement in the β-sheet structure content within the two transgenic silk fibers. The increased β-sheet content significantly enhanced the strength of the transgenic HDUY and LDUY silk fibers, a validation confirmed through subsequent mechanical performance testing. In comparison to the WT, HDUY and LDUY transgenic silk fibers exhibited increases of 39.54% and 21.45% in maximum stress, 29.79% and 16.55% in Young’s modulus, and 44.43% and 45.02% in toughness, respectively. Furthermore, the presence of low-complexity sequence regions within the Dumpy molecule contributes to its elasticity, assisting in controlling mechanical tension in the underlying epidermis (Wilkin et al., 2000). Our tensile tests demonstrated a 24.91% and 28.51% increase in elastic recovery rates for transgenic silks after cyclic stretching. These findings provided further confirmation that *Drosophila* Dumpy significantly enhanced the mechanical properties of silk, making it a promising candidate for the development of extraordinary-performance fibers. However, there is still room for further enhancement in the performance of re-Dumpy fibers when compared to chimeric spider silk fibers. Future research efforts may involve optimizing the molecular structure of the Dumpy protein, extending the length of imported *Dumpy* gene, and exploring the use of other practical silkworm strains to develop higher-performance silk materials.

In recent years, researchers have made tremendous efforts to obtain high-performance silk materials and have achieved some progress (Wen et al., 2010; Teulé et al., 2012; Heidebrecht and Scheibel, 2013). Diverse heterologous systems, including yeast, bacteria, insect cells, as well as silkworms, have been successfully applied to produce recombinant spider silk proteins, thus further expanded the sources of silk and enriched the applications of fusion silk proteins (Lazaris et al., 2002; Hauptmann et al., 2013; Jansson et al., 2016; Andersson et al., 2017; Xu et al., 2018). In addition to spider silk, various natural protein molecules with exceptional properties or unique functionalities have the potential to positively impact the mechanical performance of silk fibers through reconstruction of secondary structures during the processes of silk assembly and secretion. For example, resilin, an elastic protein commonly found in the exoskeleton structures of insects, is well-known for high strain and elasticity (Li and Daggett, 2002; Su et al., 2014). The latest research found that re-Resilin silk fibers produced by silkworm bioreactors exhibited higher fracture strength and resilience compared to WT (Zhao et al., 2023). Therefore, our findings, along with previous researches, collectively indicate that these natural protein molecules will be crucial targets for enhancing the performance of recombinant silk fibers and developing biomimetic silk materials in the future.

The large-scale production of transgenic silk fibers using domestic silkworms offers numerous advantages. Over 5,000 years of domestication have endowed domestic silkworms with traits conducive to both production and environmental safety. The limited activity range of their larvae and the loss of flight ability in adults prevent mating with wild insects, effectively avoiding the escape of foreign genes (Tomita, 2011). Furthermore, by utilizing the extracellular matrix Dumpy protein of *Drosophila* as the target protein, the resulting recombinant protein poses no threat to the environment. Silk stands out as the sole commercially produced animal silk fiber, benefitting from a well-established feeding technology. This facilitates the cost-effective and large-scale commercialization of silk fiber production through genetically modified silkworms, generating substantial economic value. Additionally, transgenic silk exhibits renewability and biodegradability, positioning it as an environmentally friendly biomaterial with potential applications in textiles, medicine, and the military (Leem et al., 2020; Zhang et al., 2023). Consequently, utilizing silkworms for large-scale transgenic silk production not only brings about economic advantages but also aligns with the trends of environmental protection and sustainable development.

## Conclusion

In summary, we focused on the biological protein, a 2.5 MDa extracellular matrix protein called Dumpy from *D. melanogaster*. We constructed two transgenic donor plasmids containing the fusion gene of *FibH-Dumpy* and *FibL-Dumpy* to express chimeric silk genes driven by endogenous fibroin heavy/light chain promoters using piggyBac-mediated transgenic technology. Two types of re-Dumpy silk were successfully obtained, and their mechanical properties including breaking strength, extensibility and elasticity, were significantly superior to those of WT silk. This study offers a new perspective on utilizing biological proteins other than spider silk protein to construct high-performance silk fibers and develop innovative biomaterials.

## Data Availability

The datasets presented in this study can be found in online repositories. The names of the repository/repositories and accession number(s) can be found below: https://www.ncbi.nlm.nih.gov/, OR224864.
