# Supplementary material for: High mechanical property silk produced by transgenic silkworms expressing the Drosophila Dumpy
Source: Front Bioeng Biotechnol. 2024 Feb 12;12:1359587. doi: 10.3389/fbioe.2024.1359587 (PMC10895422; doi:10.3389/fbioe.2024.1359587)
Supplement: Supplementary file 1 [file DataSheet1.pdf]

Supplementary Information for

High mechanical property silk produced by transgenic silkworms expressing the *Drosophila* Dumpy

Xiangping Dai[1,2], Xiaogang Ye[1,2], Liangen Shi[3], Shihua Yu[1,2], Xinqiu Wang[1,2], Boxiong Zhong[1,2]*

1 Institute of Silkworm and Bee Research, College of Animal Sciences, Zhejiang University, Hangzhou, China
2 Key Laboratory of Silkworm and Bee Resource Utilization and Innovation of Zhejiang Province, Hangzhou, China
3 Institute of Applied Bioresource Research, College of Animal Sciences, Zhejiang University, Hangzhou, China
* To whom correspondence should be addressed
E-mail: bxzhong@zju.edu.cn

This file includes:

Supporting Information

Supplementary Figures S1-S3
Supplementary Table S1-S4
Supplementary Data S1-S2

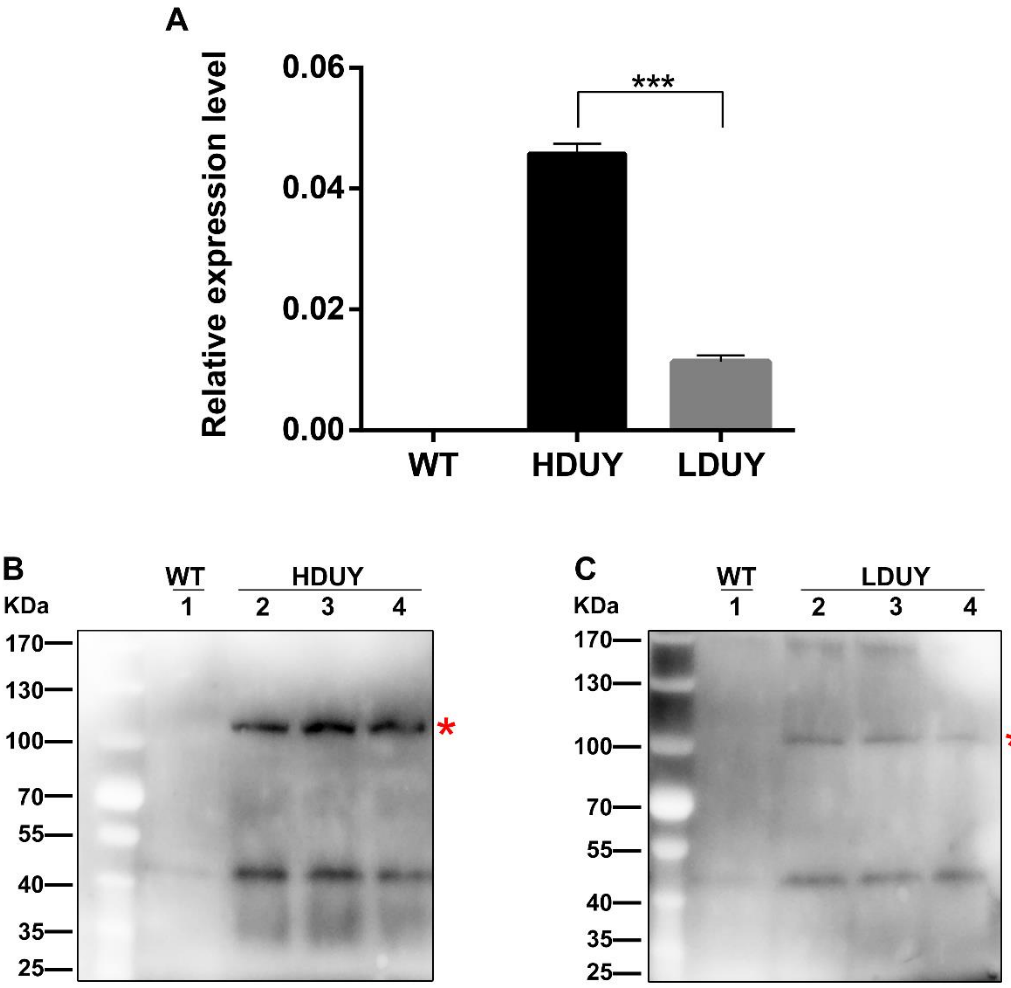

Figure S1 Expression analysis of exogenous re-Dumpy. (A) The relative gene expression levels were measured by qRT-PCR in the two transgenic lineages. Total RNA were extracted from the PSGs on the 3rd day of the fifth instar. *Bmrp49* was used as an internal control. ***$p < 0.001$. Vertical bars indicate mean ± SEM ($n = 3$). (B-C) Western blot analysis of the re-Dumpy protein extracted from cocoons. Immunoblot analysis was performed using anti-re-Dumpy antibody. The specific anti-re-Dumpy antibody was raised in rabbits against the peptides "CRPAPPPEPTQSEYV". Red asterisks indicate the predicted protein bands. The molecular masses of the protein standards are shown on the left.

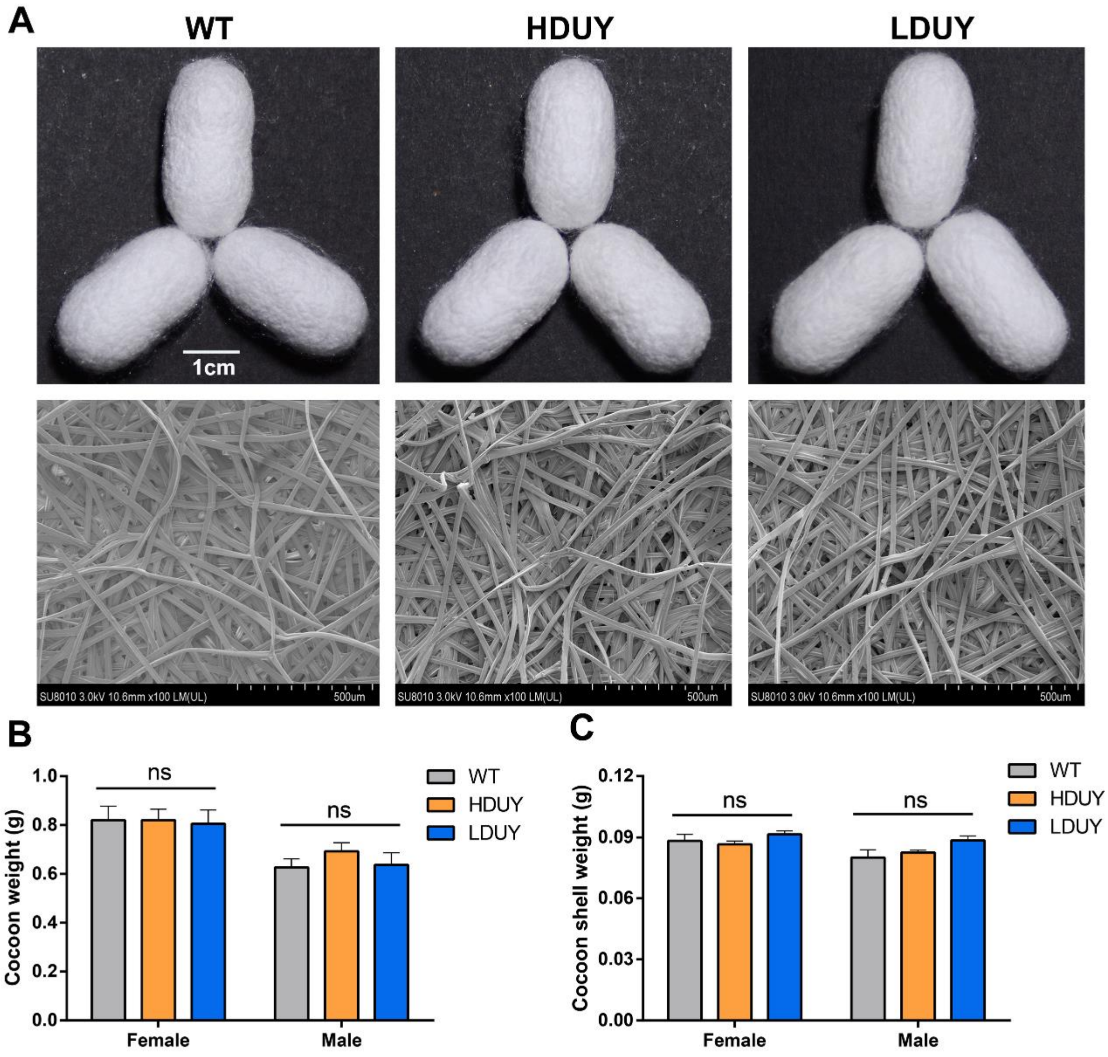

Figure S2 Silk characterization (A) Morphological observations of the cocoon shells. (B) Scanning electron microscopy (SEM) analysis of the cocoon shells out layer. (C) Cocoon weight of WT and transgenic lines (n=25). (d) Cocoon shell weight of WT and transgenic lines (n=25). Statistical analyses were performed using an independent student's t-test. n.s, $p > 0.05$.

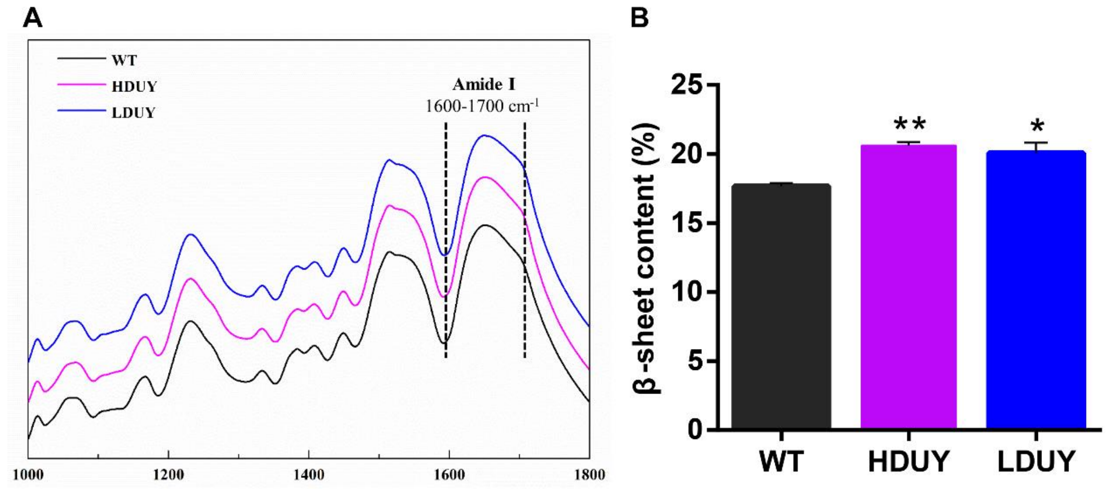

Figure S3 The analysis of β-sheet content of the transgenic cocoon silks. (A) The FTIR absorption spectra of WT, HDUY and LDUY cocoon silks at 1000-1800 cm^{-1}. (B) β-sheet content of the WT and transgenic cocoon silks. Asterisks represent significant differences with a t-test: *$p < 0.05$, **$p < 0.01$. Vertical bars indicate mean ± SEM ($n = 3$).

Table S1 List of primers used in this paper

Primer	Sequence (5'--3')	Purpose
FibHP-F	CTCGAGGTGATCAGGAAAAATGTGG	PCR for the promoter of the *FibH* gene
FibHP-R	ACTAGTCTTGAGAGTTGGAACCGAA	PCR for the promoter of the *FibH* gene
FibLP-F	CTCGAGGAATAGACCTGATAAGCG	PCR for the promoter of the *FibL* gene
FibLP-F	GTCGAC GGATTCCGTGATTAATGT	PCR for the promoter of the *FibL* gene
HDUY-F	GGACCGGTGTTACTCCCTGTGA	cloning of the *Dumpy* into the piggyBac vector
HDUY-R	CGGCTAGCTTAGTGATGGTGAT	cloning of the *Dumpy* into the piggyBac vector
LDUY-F	CCCCCCGGGGTTACTCCCTGTGA	cloning of the *Dumpy* into the piggyBac vector
LDUY-R	CGGCTAGCTTAGTGATGGTGAT	cloning of the *Dumpy* into the piggyBac vector
pBacL1-F	GACAAGCACGCCTCAGCC	inverse PCR analysis
pBacL1-R	TGAGTCAAAATGACGCATGATTATC	inverse PCR analysis
pBacL2-F	GCTCCAAGCGGCGACTG	inverse PCR analysis
pBacL2-R	GGGATGTTCTTTAGACGATGAGC	inverse PCR analysis
pBacR1-F	TCTGTATATCGAGGTTTATTTA	inverse PCR analysis
pBacR1-R	CCGATAAAAACACATGC	inverse PCR analysis
pBacR2-F	ACTCAAAATTTCTTCTAAAGTAACAA	inverse PCR analysis
pBacR2-R	CTTTAACGTACGTCACAATATG	inverse PCR analysis
Dumpy-F	GTTACTCCCTGTGAGCCAA	PCR for specific identification of the *re-Dumpy* fregment
Dumpy-R	GCAATCTCAGGCTTACATTG	PCR for specific identification of the *re-Dumpy* fregment
QDumpy-F	TCCGCCATTCAGACTGTGCTT	RT-qPCR analysis
QDumpy-R	GTTTGGTCCACAGGGTGAAGG	RT-qPCR analysis
QRp49-F	CAGGCGGTTCAAGGGTCAATAC	RT-qPCR analysis
QRp49-R	TGCTGGGCTCTTTCCACGA	RT-qPCR analysis

Table S2 Transgenic vectors microinjection and statistical results

Transgenic plasmids	Microinjected Embryos	Hatched Embryos	Hatching rate %	G1 broods	Positive G1 broods	Positive rate %
pBac[FibH-Dumpy-IE1-DsRed]	800	180	22.5	51	3	5.9
pBac[FibL-Dumpy-IE1-DsRed]	800	470	58.8	278	7	2.5

Table S3 Detection of the insertion sites of transgenic silkworms

Transgenic lineages	Chromosome	Scaffold	Insert region
HDUY-1,2,3	1	Bm_scaf142	Intron
LDUY-1,7	26	Bm_scaf25	Intron
LDUY-2,3	1	Bm_scaf8	Intergenic region
LDUY-4,5	3	Bm_scaf17	Intron
LDUY-6	10	Bm_scaf44	Intron

Table S4 Statistical results of secondary structure of cocoon silks

Strains	β-sheet (%)	helix/coils (%)	β-turn (%)
WT	17.67 ± 0.37	48.81 ± 2.43	33.52 ± 2.79
HDUY	20.62 ± 0.45	51.59 ± 2.65	27.79 ± 3.09
LDUY	20.07 ± 1.30	45.52 ± 2.67	34.41 ± 3.79

Supplementary information data
Supplementary data S1
The key component sequence of pBac[FibH-Dumpy-IE1-DsRed] expression vector
FibH promoter
FibH signal peptide
NTD and CTD of FibH
Dumpy repetitive unit
His-tag
FibH-Poly A
IE1 promoter-DsRed-SV40 poly A
GTGATCAGGAAAAATGTGGAAAGCTTAACGATTTTGTCACATTTTACTTATC
ACAACTTGTTTTTATAATAATTCGCTTAAATGAGCAGCTATTACTTAATCTCG
TAGTGGTTTTTGACAAAATCAGCTTCTTTAGAACTAAAATATCATTTTTTTCG
TAATTTTTTTAATGAAAAATGCTCTAGTGTTATACCTTTCCAAAATCACCATT
AATTAGGTAGTGTTTAAGCTTGTTGTACAAAACTGCCACACGCATTTTTTTC
TCCACTGTAGGTTGTAGTTACGCGAAAACAAAATCGTTCTGTGAAAATTCA
AACAAAAATATTTTTTCGTAAAAACACTTATCAATGAGTAAAGTAACAATTC
ATGAATAATTTCATGTAAAAAAAAAATACTAGAAAAGGAATTTTTCATTACG
AGATGCTTAAAAATCTGTTTCAAGGTAGAGATTTTTCGATATTTCGGAAAAT
TTTGTAAAACTGTAAATCCGTAAAATTTTGCTAAACATATATTGTGTTGTTTT
GGTAAGTATTGACCCAAGCTATCACCTCCTGCAGTATGTCGTGCTAATTACT
GGACACATTGTATAACAGTTCCACTGTATTGACAATAATAAAACCTCTTCAT
TGACTTGAGAATGTCTGGACAGATTTGGCTTTGTATTTTTGATTTACAAATG
TTTTTTTGGTGATTTACCCATCCAAGGCATTCTCCAGGATGGTTGTGGCATC
ACGCCGATTGGCAAACAAAAACTAAAATGAAACTAAAAAGAAACAGTTTC
CGCTGTCCCGTTCCTCTAGTGGGAGAAAGCATGAAGTAAGTTCTTTAAATAT
TACAAAAAAATTGAACGATATTATAAAATTCTTTAAAATATTAAAAGTAAGA
ACAATAAGATCAATTAAATCATAATTAATCACATTGTTCATGATCACAATTTA
ATTTACTTCATACGTTGTATTGTTATGTTAAATAAAAAGATTAATTTCTATGTA
ATTGTATCTGTACAATACAATGTGTAGATGTTTATTCTATCGAAAGTAAATAC
GTCAAAACTCGAAAATTTTCAGTATAAAAAGGTTCAACTTTTTCAAATCAG
CATCAGTTCGGTTCCAACTCTCAAGACTAGTATGAGAGTCAAAACCTTTGT
GATCTTGTGCTGCGCTCTGCAGTATGTCGCTTATACAAATGCAAACATCAAT
GATTTTGATGAGGACTATTTTGGGAGTGATGTCACTGTCCAAAGTAGTAATA
CAACAGATGAAATAATTAGAGATGCATCTGGGGCAGTTATCGAAGAACAAA
TTACAACTAAAAAAATGCAACGGAAAAATAAAAATCATGGAATACTTGGAA
AAAATGAAAAAATGATCAAGACGTTCGTTATAACCACGGATTCCGACGGTA
ACGAGTCCATTGTAGAGGAAGATGTGCTCATGAAGACACTTTCCGATGGTA
CTGTTGCTCAAAGTTATGTTGCTGCTGATGCGGGAGCATATTCTCAGAGCG
GGCCATACGTATCAAACAGTGGATACAGCACTCATCAAGGATATACGAGCG
ATTTCAGCACTAGCGCTGCAGTCGGTGCCGGTGCTGGTTCAGGTGCTGGTG
CTGGTTCAGGTGCCGGTGCTGGTGGTAGCGTCAGTTACGGAGCTGGCAGG
GGATACGGACAAGGTGCAGGAAGTGCAGCTTCCTCTGTGTCATCTGCTTCA
TCTCGCAGTTACGACTATTCTCGTCGTAACGTCCGCAAAAACTGTGGAATT

CCTAGAAGACAACTAGCTGTTAAATTCAGAGCACTGCCTTGTGTGAATTGC
ACCGGTGTTACTCCCTGTGAGCCAAATCCATGTGGTTCTAATGCTGTTTGCC
GCGAACGCAATGGAATTGGTTCATGTCAATGCTTGCCCGACCACTTTGGTG
ATCCATACCAAAGCTGCCGCCCCGAATGTGTCCGCCATTCAGACTGTGCTT
CAAACAAGGCTTGTCAACAACAAAAGTGTCGCGACCCCTGCCCCGGTACT
TGTGGTTCAAATGCTGATTGCAGCGTTACTAATCATTTACCAACTTGCACTT
GCCGCATTGGTTACACTGGAGATCCATACCGCTACTGCCATGTTGAACCCCC
ACAACTGCCTGCTCGCGTTACTGAACCATCACAACCATGTCGCCCTTCACC
CTGTGGACCAAACAGTCAATGTCGCGAACTAAATGGTCAAGCTGTTTGCAG
TTGTCTGGAGTTATATATTGGTCTGCCACCAAACTGTCGCCCTGAATGTGTG
TTGTCAACTGAATGCCCTACTGACAAGGCTTGTATTTCACAGCGCTGCCAA
GACCCATGCCCTGGTACTTGTGGTATCAATGCTGAGTGCCGCGTCAGAAAT
CATTCACCTCTATGCCAATGCAGACAAGGATTTACTGGTGATAGCTTCACTA
GATGTTATCCCCTTCCACCTCCCCCACCTGTAATTGAAAGAGTTGAAAGAG
ACCCATGCCTGCCCTCACCTTGTGGTTTGAATTCACAATGTAGGAATGTTCA
AGGTGTTCCATCATGCACCTGTCTGCCTGACTTTCTGGGTGCTCCACCAAAT
TGCAGACCTGAATGTACTATCTCAGCTGAATGTCCATCAAACCTGGCTTGTA
TCAGAGAAAGATGCATTGACCCATGCCCTGGATCTTGTGGATATGCTGCTG
AATGCTCAGTTGTTAATCACACCCCAATCTGTGTTTGCCCTGCTGGATTTAC
TGGAGATCCTTTCTCATCGTGTAGACCTGCTCCACCACCTGAACCAACACA
ATCAGAATATGTTGATCCATGCAACCCTTCACCATGTGGTCCAAATGCTCAG
TGTAATGCTGGTATTTGCACTTGCCTAGCAGAATTTCATGGTGACCCTTACT
CTGGTTGTAGACCTGAATGTGTGCTTAACTCAGACTGCCCAAGAGACAAA
GCTTGTCACTCATCAAAATGTGTTAATCCATGCCCTGGTACCTGTGGTGAAA
ATGCTATCTGTGATGTTATAAATCATATTCCAATGTGTAGATGCCCTGAAAGA
ACAGCTGGTTCAGCTTTTATAAGATGCTCACCTGTTCAAATCACTGTTAGCA
ATCCATGTAGACCTTCACCATGTGGTCCCAACTCACAATGTAGAGAGGTAA
ATCAACAAGCTGTCTGCTCGTGTTTGCCATCATTCATAGGAGCCCCTCCTTC
TTGCAGACCTGAATGTACTTCAAATTCGGAGTGTGCACCAACTCAAGCTTG
CCTGAACCAAAGATGTGGTGATCCATGTCCTGGCACTTGTGGTGTTGGTGC
TAATTGTGCTGTTGTTTCACACTCACCATTTTGCACTTGTCCTGAAAGATTC
ACTGGTAACCCATTCATTAGATGTCAACCACAAATTGAACCACCTGTTAGA
GATGTTGCTCCTGTTGACCCATGTAGGCCTTCACCATGTGGACCATATTCAC
AGTGCAGACCTGTTGGTGAAGCTCCTGCTTGCAGCTGTGTTGAGACTTACA
TTGGTAGACCCCCAAACTGCAGACCTGAATGTGTTACCTCTTCAGATTGCT
CATCACAACTGGCATGTGTTAATCAGAAGTGTGTGGATCCTTGCCCTGGCA
GATGTGGTCTGAATGCTGAATGCTTTGTTGTCTCACATGCTGTGCAATGCAT
CTGCCAACAAGGTTTTAATGGAGATCCTTTTGTTCAATGTAAGCCTGAGATT
GCTTATGAAAATGAAATCCATCACCATCACCATCACTAAGCTAGCTTTTTAA
TATAAAATAACCCTTGTTTCTTACTTCGTCCTGGATACATCTATGTTTTTTTT
TCGTTAATAAATGAGAGCATTTAAGTTATTGTTTTTAATTACTTTTTTTTAGA
AAACAGATTTCGGATTTTTTGTATGCATTTTATTTGAATGTACTAATATAATCA
ATTAATCAATGAATTCATTTATTTAAGGGATAACAATAATCCATGAATTCACA
TGCACATTTAAAACAAAACTAAATTACAATAGGTTCATATAAAAACAACAA

GTATGCCTTCTCAACTAAGAATACTATATTGTTTAAACCGTAAAAAAAGTCA
TATTTCTGTATATCAAAACACATCTAATATTAAAAAAACAGTCAGCAAGCAC
TTACAAGTGTGGGCTCGGACAGCAATTACCTGGTCTCAGGAGAATGTGCCA
CACTTGCAGCTCTGCTTGTGTGCGCGTTACCACAAATCCCAACGGCGCAGT
GTACTTGTTGTATGTAAATAAATCTCGATAAAGGCGCGGCGCGCGAATGCA
GCTGATCACGTACGCTCCTCGTGTCCCGTTCAAGGACGGTGTTATCGACCT
CAGATTAATATTTATCGGCCGACTGTTTTCGTATCCGCTCACCAAACGTGTTT
TTGCATTAACATTGTATGTCGGCGGATGTTCTGTATCTAATTTGAATAAATAA
ATGATAACCGCATTGGTTTTAGAGGGCATAATAAAAAAAATATTATTATCGTG
TTCGCCATTAGGGCAGTATAAATTGACGTTCATGTTGAATATTGTTTCAGTTG
CAAGTTGACATTGGCGGCGACACGATCGTGAACAACCAAACGACTATGGT
GCGCTCCTCCAAGAACGTCATCAAGGAGTTCATGCGCTTCAAGGTGCGCAT
GGAGGGCACCGTGAACGGCCACGAGTTCGAGATCGAGGGCGAGGGCGAG
GGCCGCCCCTACGAGGGCCACAACACCGTGAAGCTGAAGGTGACCAAGG
GCGGCCCCCTGCCCTTCGCCTGGGACATCCTGTCCCCCCAGTTCCAGTACG
GCTCCAAGGTGTACGTGAAGCACCCCGCCGACATCCCCGACTACAAGAAG
CTGTCCTTCCCCGAGGGCTTCAAGTGGGAGCGCGTGATGAACTTCGAGGA
CGGCGGCGTGGTGACCGTGACCCAAGACTCCTCCCTGCAGGACGGCTGCT
TCATCTACAAGGTGAAGTTCATCGGCGTGAACTTCCCCTCCGACGGCCCCG
TAATGCAGAAGAAGACCATGGGCTGGGAGGCCTCCACCGAGCGCCTGTAC
CCCCGCGACGGCGTGCTGAAGGGCGAGATCCACAAGGCCCTGAAGCTGAA
GGACGGCGGCCACTACCTGGTGGAGTTCAAGTCCATCTACATGGCCAAGA
AGCCCGTGCAGCTGCCCGGCTACTACTACGTGGACTCCAAGCTGGACATCA
CCTCCCACAACGAGGACTACACCATCGTGGAGCAGTACGAGCGCACCGAG
GGCCGCCACCACCTGTTCCTGTAGTTGTTGTTGTTAACTTGTTTATTGCAGC
TTATAATGGTTACAAATAAAGCAATAGCATCACAAATTTCACAAATAAAGCA
TTTTTTTCACTGCATTCTAGTTGTGGTTTGTCCAAACTCATCAATGTATC

Supplementary data S2

The key component sequence of pBac[FibL-Dumpy-IE1-DsRed] expression vector

FibL promoter

FibL signal peptide

FibL CDS

Dumpy repetitive unit

His-tag

FibL-Poly A

IE1 promoter-DsRed-SV40 poly A

GAATAGACCTGATAAGCGTATGTAACATTCAGACGAAATCAAATAACAAAG
TGGTGCCTATCCCACTTTTTTTGATCCAGACAAAGAAAATAAGTGTTTTCGG
TGAGTTGAAAAATTAATTTCAGGAAACAACAAAAATAATGACGCAAAAGT
ACACCGGAGTGAAAATTAAAACTAAGAAAGTAATCGCTAAAAATTATTCAT
CTCGTGAATTGATTGAGCGCGATAATAACGCAGTACTATTGGAGAGATTCTA
TGTTTAATATATTAATGATATGATATAAAAAAGGGTGCGTGTACTTATCTACG
CGCGTAAGAAGTTATACTTTATTTTCATTAAATTTATTTCTTTTTTTTTATTTC
AATTTTAATCAATTTTAAAAAAAATCGAATAAACAACATCCTCAAACACGC
ATATTGGACATCCCTTTTCTTGACATCGTATAAATTCGGTAATTCTCGGTACG
GTTCGGAAAGTGCACCTGCGGCTATATTCAGACTCGCCAAGTTACGTCAGT
CGTATTGTAATGAGCGATTTAGTGGGCAACTTCATTCTGTTAATTTTGTGTCA
CGGTGCGCGCGCATCGTAAAATTTCACTCTCATAGATTTTTCATAACGTGCC
TAAAGAAGTATAACTTCAATAATTTAAATTAAAAAAAAAAACATGCATAGA
ATAATTATATGAATTATTTAAAATGTCATTTACCGACATTGACATAACAGACG
ACGTTAACACTACAAAACATTTTAATTCCACATTGTTACATATTCAACAGTT
AAATTTGCGTTAATTCTCGATGCGAACAAATATAAGAACAATCGGATCAATT
AGATCGCTTTGTTTCGAGCAACACTTAGTTTAACTAGAGGCGTACACCTCA
AGAAATCATCTTCATTAGAAACTAAACCTTAAAATCGCAATAATAAAGCATA
GTCAATTTTAACTGAAATGCAAAATCTTTTGAACGTTAGATGCTGTCAGCGT
TCGTTGGTACAGTTGTTTGATATTTATTTTAATTGTCTTTTTATATATAAATAGT
GGAACATTAATCACGGAATCCGTCGACATGAAGCCTATATTTTTGGTATTAC
TCGTCGCTACAAGCGCCTACGCTGCACCATCGGTGACCATCAATCAATACA
GTGATAATGAAATTCCACGCGACATTGATGATGGAAAAGCTAGTTCCGTAAT
CTCACGTGCATGGGACTACGTCGATGACACTGACAAAAGCATCGCCATCCT
CAACGTTCAAGAGATATTGAAGGACATGGCCAGCCAGGGCGATTATGCAAG
TCAAGCATCAGCGGTGGCCCAAACCGCCGGAATTATCGCCCATCTATCTGC
CGGTATCCCCGGTGATGCCTGTGCAGCCGCTAACGTCATTAACTCTTACACA
GACGGCGTCAGGTCCGGAAACTTCGCGGGCTTCAGACAATCTCTCGGTCC
CTTCTTCGGACACGTGGGACAAAACTTGAATCTTATCAATCAACTCGTCATC
AACCCTGGTCAACTCCGATACTCTGTCGGACCAGCCCTGGGTTGTGCCGGA
GGTGGAAGAATCTATGACTTCGAAGCCGCTTGGGATGCAATCTTAGCCAGC
AGTGACTCTAGTTTCTTAAATGAAGAGTACTGCATCGTCAAGAGATTGTAC
AACTCTCGCAACAGCCAAAGCAACAACATCGCTGCCTACATCACCGCTCAC
TTACTTCCCCCGGTTGCTCAAGTGTTCCACCAATCAGCTGGATCAATCACA
GACCTCCTGAGAGGCGTTGGCAACGGTAATGACGCGACCGGTTTAGTTGCT

AATGCTCAAAGATATATTGCACAAGCAGCCAGCCAGGTTCACGTCCCCGGG
GTTACTCCCTGTGAGCCAAATCCATGTGGTTCTAATGCTGTTTGCCGCGAAC
GCAATGGAATTGGTTCATGTCAATGCTTGCCCGACCACTTTGGTGATCCATA
CCAAAGCTGCCGCCCCGAATGTGTCCGCCATTCAGACTGTGCTTCAAACAA
GGCTTGTCAACAACAAAAGTGTCGCGACCCCTGCCCCGGTACTTGTGGTTC
AAATGCTGATTGCAGCGTTACTAATCATTTACCAACTTGCACTTGCCGCATT
GGTTACACTGGAGATCCATACCGCTACTGCCATGTTGAACCCCCACAACTG
CCTGCTCGCGTTACTGAACCATCACAACCATGTCGCCCTTCACCCTGTGGA
CCAAACAGTCAATGTCGCGAACTAAATGGTCAAGCTGTTTGCAGTTGTCTG
GAGTTATATATTGGTCTGCCACCAAACTGTCGCCCTGAATGTGTGTTGTCAA
CTGAATGCCCTACTGACAAGGCTTGTATTTCACAGCGCTGCCAAGACCCAT
GCCCTGGTACTTGTGGTATCAATGCTGAGTGCCGCGTCAGAAATCATTCAC
CTCTATGCCAATGCAGACAAGGATTTACTGGTGATAGCTTCACTAGATGTTA
TCCCCTTCCACCTCCCCCACCTGTAATTGAAAGAGTTGAAAGAGACCCATG
CCTGCCCTCACCTTGTGGTTTGAATTCACAATGTAGGAATGTTCAAGGTGTT
CCATCATGCACCTGTCTGCCTGACTTTCTGGGTGCTCCACCAAATTGCAGA
CCTGAATGTACTATCTCAGCTGAATGTCCATCAAACCTGGCTTGTATCAGAG
AAAGATGCATTGACCCATGCCCTGGATCTTGTGGATATGCTGCTGAATGCTC
AGTTGTTAATCACACCCCAATCTGTGTTTGCCCTGCTGGATTTACTGGAGAT
CCTTTCTCATCGTGTAGACCTGCTCCACCACCTGAACCAACACAATCAGAA
TATGTTGATCCATGCAACCCTTCACCATGTGGTCCAAATGCTCAGTGTAATG
CTGGTATTTGCACTTGCCTAGCAGAATTTCATGGTGACCCTTACTCTGGTTG
TAGACCTGAATGTGTGCTTAACTCAGACTGCCCAAGAGACAAAGCTTGTCA
CTCATCAAAATGTGTTAATCCATGCCCTGGTACCTGTGGTGAAAATGCTATC
TGTGATGTTATAAATCATATTCCAATGTGTAGATGCCCTGAAAGAACAGCTG
GTTCAGCTTTTATAAGATGCTCACCTGTTCAAATCACTGTTAGCAATCCATG
TAGACCTTCACCATGTGGTCCCAACTCACAATGTAGAGAGGTAAATCAACA
AGCTGTCTGCTCGTGTTTGCCATCATTCATAGGAGCCCCTCCTTCTTGCAGA
CCTGAATGTACTTCAAATTCGGAGTGTGCACCAACTCAAGCTTGCCTGAAC
CAAAGATGTGGTGATCCATGTCCTGGCACTTGTGGTGTTGGTGCTAATTGTG
CTGTTGTTTCACACTCACCATTTTGCACTTGTCCTGAAAGATTCACTGGTAA
CCCATTCATTAGATGTCAACCACAAATTGAACCACCTGTTAGAGATGTTGCT
CCTGTTGACCCATGTAGGCCTTCACCATGTGGACCATATTCACAGTGCAGA
CCTGTTGGTGAAGCTCCTGCTTGCAGCTGTGTTGAGACTTACATTGGTAGA
CCCCCAAACTGCAGACCTGAATGTGTTACCTCTTCAGATTGCTCATCACAA
CTGGCATGTGTTAATCAGAAGTGTGTGGATCCTTGCCCTGGCAGATGTGGT
CTGAATGCTGAATGCTTTGTTGTCTCACATGCTGTGCAATGCATCTGCCAAC
AAGGTTTTAATGGAGATCCTTTTGTTCAATGTAAGCCTGAGATTGCTTATGA
AAATGAAATCCATCACCATCACCATCACTAAGCTAGCATAAGAACTGTAAAT
AATGTATATATATAATTATATAAAAGATATATATAAACCATATACAAACATATAT
ATATCATTATAAGACAATCTACCTATATAAAAACAGACTAAAATTAATAATTA
TGTATACTTTAATTGTGTTTAGGACATTTTATGCAAATTGTGTTTGCGTTAGG
ATTTTTTTTGGAAGTTTTTTAGATTATTTATGAATATATAAATAAATATACGTT
AATATAATATATATTATATAAATCAACGACACGGCTTTTCATTTTGGTGATGAT

CAATCTTATTGTTCTTCTAATTGATTTTTTTGTACAATAAAGATGTATCCAGTT
TTCCAGATAATGTGCCACACTTGCAGCTCTGCTTGTGTGCGCGTTACCACA
AATCCCAACGGCGCAGTGTACTTGTTGTATGTAAATAAATCTCGATAAAGGC
GCGGCGCGCGAATGCAGCTGATCACGTACGCTCCTCGTGTCCCGTTCAAGG
ACGGTGTTATCGACCTCAGATTAATATTTATCGGCCGACTGTTTTCGTATCCG
CTCACCAAACGTGTTTTTGCATTAACATTGTATGTCGGCGGATGTTCTGTAT
CTAATTTGAATAAATAAATGATAACCGCATTGGTTTTAGAGGGCATAATAAA
AAAAATATTATTATCGTGTTCGCCATTAGGGCAGTATAAATTGACGTTCATGT
TGAATATTGTTTCAGTTGCAAGTTGACATTGGCGGCGACACGATCGTGAAC
AACCAAACGACTATGGTGCGCTCCTCCAAGAACGTCATCAAGGAGTTCATG
CGCTTCAAGGTGCGCATGGAGGGCACCGTGAACGGCCACGAGTTCGAGAT
CGAGGGCGAGGGCGAGGGCCGCCCCTACGAGGGCCACAACACCGTGAAG
CTGAAGGTGACCAAGGGCGGCCCCCTGCCCTTCGCCTGGGACATCCTGTC
CCCCCAGTTCCAGTACGGCTCCAAGGTGTACGTGAAGCACCCCGCCGACAT
CCCCGACTACAAGAAGCTGTCCTTCCCCGAGGGCTTCAAGTGGGAGCGCG
TGATGAACTTCGAGGACGGCGGCGTGGTGACCGTGACCCAAGACTCCTCC
CTGCAGGACGGCTGCTTCATCTACAAGGTGAAGTTCATCGGCGTGAACTTC
CCCTCCGACGGCCCCGTAATGCAGAAGAAGACCATGGGCTGGGAGGCCTC
CACCGAGCGCCTGTACCCCCGCGACGGCGTGCTGAAGGGCGAGATCCACA
AGGCCCTGAAGCTGAAGGACGGCGGCCACTACCTGGTGGAGTTCAAGTCC
ATCTACATGGCCAAGAAGCCCGTGCAGCTGCCCGGCTACTACTACGTGGAC
TCCAAGCTGGACATCACCTCCCACAACGAGGACTACACCATCGTGGAGCA
GTACGAGCGCACCGAGGGCCGCCACCACCTGTTCCTGTAGTTGTTGTTGTT
AACTTGTTTATTGCAGCTTATAATGGTTACAAATAAAGCAATAGCATCACAA
ATTTCACAAATAAAGCATTTTTTTCACTGCATTCTAGTTGTGGTTTGTCCAA
ACTCATCAATGTATC
